# Effect of Metronidazole on the Growth of Vaginal Lactobacilli *in vitro*

**DOI:** 10.1155/S1064744901000072

**Published:** 2001

**Authors:** Jose A. Simoes, Alla A. Aroutcheva, Susan Shott, Sebastian Faro

**Affiliations:** 1 Department of Obstetrics and Gynecology Rush-Presbyterian-St. Luke’sMedical Center 1653 W. Congress Pkwy 720 Pav. Chicago IL 60612 USA; 2 Department of Obstetrics and Gynecology State University of Campinas (Unicamp) Sao Paulo Brazil

## Abstract

*Objective:* To determine whether metronidazole has an adverse effect on the growth of *Lactobacillus*.

*Methods:* Hydrogen peroxide- and bacteriocin-producing strains of *Lactobacillus* were used as test strains. Concentrations of metronidazole used ranged from 128 to 7000 μg/ml. Susceptibility to metronidazole was conducted by the broth microdilution method recommended by the National Committee for Clinical Laboratory Standards.

*Results:* Growth of *Lactobacillus* was partially inhibited at concentrations between 1000 and 4000 μg/ml (*p* = 0.014). Concentrations ≥ 5000 μg/ml completely inhibited growth of *Lactobacillus*. Concentrations between 128 and 256 μg/ml stimulated growth of *Lactobacillus* (*p* = 0.025 and 0.005, respectively). Concentrations of metronidazole between 64 and 128 μg/ml or ≥ 512 μg/ml did not have an inhibitory or a stimulatory effect on the growth of *Lactobacillus* compared to the control.

*Conclusions:* High concentration of metronidazole, i.e. between 1000 and 4000 μg/ml, partially inhibited the growth of *Lactobacillus*. Concentrations ≥ 5000 μg/ml completely suppressed the growth of *Lactobacillus*. Concentrations between ≥ 128 and ≤ 256 μg/ml stimulated the growth of *Lactobacillus*. Further investigation to determine the ideal concentration of metronidazole is needed in order to use the antimicrobial agent effectively in the treatment of bacterial vaginosis.


Infect Dis Obstet Gynecol 2001;9:41–45

### Effect of metronidazole on the growth of vaginal lactobacilli *in vitro*

**Jose A. Simoes[1,2], Alla A. Aroutcheva[1], Susan Shott[1] and Sebastian Faro[1]**

[1]*Department of Obstetrics and Gynecology, Rush-Presbyterian-St. Luke's Medical Center, Chicago, IL, USA*
[2]*Department of Obstetrics and Gynecology, State University of Campinas (Unicamp), Sao Paulo, Brazil*

***Objective:*** To determine whether metronidazole has an adverse effect on the growth of *Lactobacillus*.
***Methods:*** Hydrogen peroxide- and bacteriocin-producing strains of *Lactobacillus* were used as test strains. Concentrations of metronidazole used ranged from 128 to 7000 μg/ml. Susceptibility to metronidazole was conducted by the broth microdilution method recommended by the National Committee for Clinical Laboratory Standards.
***Results:*** Growth of *Lactobacillus* was partially inhibited at concentrations between 1000 and 4000 μg/ml ($p = 0.014$). Concentrations ≥ 5000 μg/ml completely inhibited growth of *Lactobacillus*. Concentrations between 128 and 256 μg/ml stimulated growth of *Lactobacillus* ($p = 0.025$ and 0.005, respectively). Concentrations of metronidazole between 64 and 128 μg/ml or ≥ 512 μg/ml did not have an inhibitory or a stimulatory effect on the growth of *Lactobacillus* compared to the control.
***Conclusions:*** High concentration of metronidazole, i.e. between 1000 and 4000 μg/ml, partially inhibited the growth of *Lactobacillus*. Concentrations ≥ 5000 μg/ml completely suppressed the growth of *Lactobacillus*. Concentrations between ≥ 128 and ≤ 256 μg/ml stimulated the growth of *Lactobacillus*. Further investigation to determine the ideal concentration of metronidazole is needed in order to use the antimicrobial agent effectively in the treatment of bacterial vaginosis.

Key words: METRONIDAZOLE; *LACTOBACILLUS*; BACTERIAL VAGINOSIS

Bacterial vaginosis (BV) is a clinical syndrome of unknown etiology. It occurs when normal vaginal flora is replaced by an overgrowth of *Gardnerella vaginalis* and anaerobic microorganisms[1]. The current therapeutic goal for BV is to reestablish the normal vaginal flora[2]. Metronidazole, orally or intravaginally, is the drug of choice recommended by the Centers for Disease Control and Prevention for treatment of BV[2]. However, after 1 month, cure rates for both treatment regimens range from 60% to 70%. These high failure rates seem to occur because of an inability to reestablish the lactobacilli-predominant vaginal flora after treatment[3]. In a recent article, Paavonen and colleagues[4] compared oral metronidazole to 3 days of clindamycin ovules and achieved a 68% cure race[4]. Thus neither metronidazole nor clindamycin appears to be effective treatment for BV.

It is well established that metronidazole is significantly active against anaerobes but is

Correspondence to: Jose A. Simoes, MD, PhD, Department of Obstetrics and Gynecology, Rush-Presbyterian-St. Luke's Medical Center, 1653 W. Congress Pkwy, 720 Pav., Chicago, IL 60612, USA

not active against *G. vaginalis* and facultative anaerobes[5]. There are relatively few published data on the effects of metronidazole on vaginal lactobacilli. These published studies suggest that metronidazole has no effect on lactobacilli[6,7]. This study was conducted to assess the *in vitro* effect of different metronidazole concentrations on the growth of vaginal lactobacilli.

### MATERIALS AND METHODS

Metronidazole activity was evaluated, *in vitro,* against eight clinical strains of vaginal *Lactobacillus.* These strains were recovered from women with healthy vaginal microflora. Lactobacilli were initially identified on the basis of the colony morphology when grown on Mann–Rogosa–Sharp (MRS) agar and the morphologic appearance on Gram stain. A MicroLog Microbial Identification System® (Biolog Inc., Hayward, CA) was used to identify the following species: *Lactobacillus casei* (four strains), *L. acidophilus* (three strains) and *L. jensenii* (one strain). These bacteria were maintained at –70°C in skim milk (Difco Laboratories, Detroit, MI) prior to testing.

Susceptibility to metronidazole was determined by the broth microdilution method recommended by the National Committee for Clinical Laboratory Standards (NCCLS)[8]. MRS broth (Difco, Becton Dickinson Microbiology Systems, Sparks, MD) was prepared for use in this study. Fresh subcultures of lactobacilli were used after overnight growth on an MRS agar plate under anaerobic conditions. The inoculum was prepared by suspending several of these colonies in sterile phosphate-buffered saline (pH 7.2) to achieve a turbidity of 0.5 McFarland standard, determined by nephelometry. This resulted in a suspension containing approximately 1–2 × $10^8$ CFU/ml. These suspensions were further diluted with MRS broth to obtain a final inoculum suspension of 5–10 × $10^5$ CFU/ml. They were then dispensed to sterile microdilution test plates (Honeycomb Microwell Plate®; Labsystems, Finland) prepared with different concentrations of metronidazole (Sigma Chemical Co., St. Louis, MO). After the addition of *Lactobacillus* inocula, the final range of metronidazole concentrations was 1–7000 µg/ml. The plates were overlaid with sterile paraffin oil and incubated at 36°C in a Bioscreen C Analyser System® (Labsystems) for 48 hours.

The optical density of each tested sample was measured automatically at 4-h intervals on a wide band. Statistical analyses were performed by the Freidman test. The Mann–Whitney test was used to compare the species of lactobacilli with respect to the percentage of growth inhibition at different concentrations of metronidazole.

### RESULTS

The growth of *Lactobacillus* in the presence of metronidazole depended on the concentration of metronidazole. Growth was stimulated at concentrations between 128 and 256 µg/ml ($p$ = 0.025 and 0.005, respectively; Figure 1). No statistically significant differences were found between the control and metronidazole concentrations of 512 µg/ml or ≤ 64 µg/ml.

Concentrations of 1000–4000 µg/ml had a partial inhibition of growth ($p$ = 0.014; Figure 2). Concentrations of metronidazole ≥ 5000 µg/ml showed complete inhibition of *Lactobacillus* growth. The inhibitory effect of metronidazole started at a concentration of 1000 µg/ml and was more intense at the higher concentrations (Table 1). There was a statistically significantly greater percentage of growth inhibition for *L. casei* strains compared to *L. acidophilus* strains for a

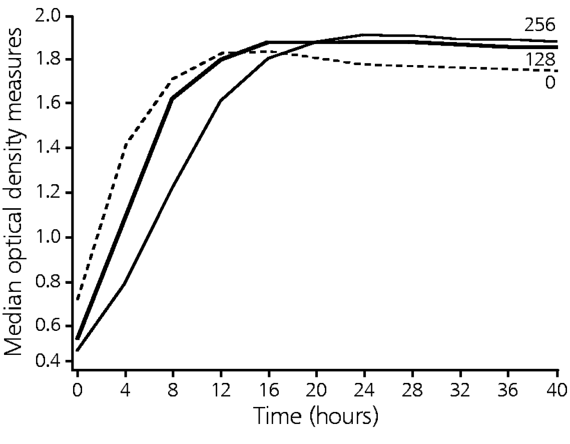

**Figure 1** Median *Lactobacillus* growth in metronidazole concentrations of 128 and 256 µg/ml metronidazole concentration of 4000 μg/ml (83.0 ± 11.0 *vs* 64.0 ± 4.5; $p =$ 0.034).

Table 2 depicts the effect of varying metronidazole concentrations on the species of *Lactobacillus* tested. Concentrations ≥ 4000 μg/ml were similar in their inhibitory effect on the growth of *Lactobacillus*. Concentrations ≥ 3000 μg/ml were also similar, except for *L. acidophilus* strain 117, which did not appear to be affected to the same degree as the other strains and species.

### DISCUSSION

This study demonstrates that different metronidazole concentrations can have a varied effect on the growth of lactobacilli *in vitro*. Concentrations < 512 μg/ml have a tendency to stimulate growth.

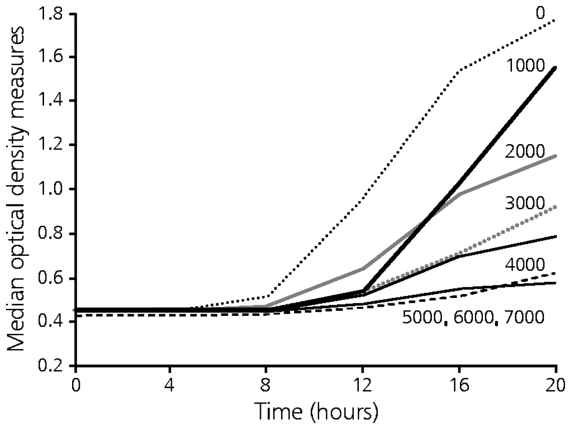

**Figure 2** Median *Lactobacillus* growth in metronidazole concentrations ranging from 1000 to 7000 μg/ml

However, at concentrations ≥ 1000 μg/ml, growth is inhibited. These findings may be pertinent to the current treatment of BV, particularly with metronidazole intravaginal treatment.

The recommended regimens include metronidazole 500 mg orally, twice per day for 7 days, and metronidazole gel 0.75% (7.5 mg/g), one full applicator (5 g, containing 37.51 mg of metronidazole) intravaginally twice per day for 5 days[2]. Cure rates 7–10 days after the oral regimen are 84% and after the vaginal regimen are 75%. After 1 month, however, the cure rates after both treatment regimens are only 60–70%, and the BV recurrence rate is up to 20% after treatment[3,9,10]. The reasons for the recurrence are not understood. One possible explanation is the failure to reestablish the normal, and perhaps protective, *Lactobacillus*-predominant vaginal flora following therapy[5].

Metronidazole, being primarily effective against obligate anaerobic bacteria, is thought to have little

**Table 1** *Lactobacillus* optical density for different metronidazole concentrations (after 24 h)

| *Metronidazole concentration (μg/ml)* | *Optical density* | |
|---|---|---|
| | *Mean* | *Standard deviation* |
| 0 (control) | 1.66 | 0.38 |
| 128 | 1.67 | 0.45 |
| 256 | 1.64 | 0.49 |
| 1000 | 1.28 | 0.50 |
| 2000 | 1.02 | 0.40 |
| 3000 | 0.86 | 0.33 |
| 4000 | 0.70 | 0.32 |

**Table 2** Percentage of growth inhibition of *Lactobacilli* by high concentrations of metronidazole (after 24 h)

| | *Metronidazole concentration (μg/ml)* | | | | | | |
|---|---|---|---|---|---|---|---|
| *Clinical isolate* | *7000* | *6000* | *5000* | *4000* | *3000* | *2000* | *1000* |
| *L. acidophilus* (29) | 91.1 | 87.0 | 88.6 | 66.7 | 76.9 | 58.8 | 29.0 |
| *L. acidophilus* (117) | 86.1 | 86.0 | 70.3 | 58.8 | 35.4 | 19.8 | 15.4 |
| *L. acidophilus* (160) | 95.1 | 93.1 | 84.2 | 66.5 | 61.0 | 37.6 | 13.2 |
| *L. casei (30)* | 98.9 | 98.9 | 96.9 | 96.6 | 90.0 | 90.7 | 84.7 |
| *L. casei (102)* | 99.8 | 98.5 | 94.8 | 76.2 | 73.6 | 48.5 | 20.8 |
| *L. casei* (66) | 85.6 | 86.7 | 73.0 | 72.2 | 57.0 | 57.1 | 23.2 |
| *L. casei (130)* | 89.5 | 96.7 | 93.9 | 86.8 | 84.4 | 65.5 | 30.0 |
| *L. jensenii* (135) | 92.9 | 84.6 | 82.7 | 75.2 | 64.8 | 54.8 | 21.7 |

effect on the growth of the normal vaginal flora[11]. The available data regarding the effect of metronidazole on the growth of vaginal lactobacilli suggest that metronidazole would be most likely to preserve endogenous lactobacilli. Agnew and Hillier[6] found that treatment of women with BV using oral or vaginal metronidazole led to increased colonization by lactobacilli. However, they also found that about half of the women lacked vaginal lactobacilli $H_2O_2$ producers following treatment with metronidazole.

Another study showed that intravaginal metronidazole gel 0.75% does not inhibit lactobacilli[7]. However, the authors recovered lactobacilli from only 65% of the women 1 month after treatment. In the study performed by Bayer and colleagues[12], metronidazole was totally ineffective against the lactobacilli. However, the maximal concentration they tested was 320 μg/ml, based on the generally achievable serum concentration of 12.5 μg/ml after the administration of oral metronidazole.

After vaginal administration of 37.5 mg (a 5 g applicator dose) of 0.75% metronidazole gel, the maximal serum concentration was 0.2 μg/ml[13], whereas vaginal concentrations of the drug may reach levels of 1000 μg/ml (Curatek Pharmaceuticals, Elk Grove, IL). Little information on vaginal concentration after oral metronidazole dosing is available. However, one study found a vaginal concentration of only 26 μg/ml 6 h after a 2 g oral dose[14].

This study demonstrates that high concentrations ($\geq$ 1000 μg/ml) of metronidazole can inhibit the growth of vaginal lactobacilli *in vitro*. It is important to determine the lowest effective dose of vaginal metronidazole against BV in order to reduce the incidence of side-effects[15]. Livengood and colleagues[16] recently showed that once-per-day dosing of 0.75% metronidazole gel has an efficacy equivalent to that of the currently used twice-per-day dosing in the treatment of BV. These authors suggested that such modification would improve the regimen by decreasing the total amount of metronidazole required. Further studies are needed to determine whether lower vaginal doses of $\leq$ 512 μg/ml are efficacious in treating BV and restoring *Lactobacillus* to a dominant role.

### REFERENCES

1. Ugwumadu AHN, Hay P. Bacterial vaginosis: sequelae and management. *Curr Opin Infect Dis* 1999;12:53–9
2. Centers for Disease Control and Prevention. 1998 Guidelines for treatment of sexually transmitted diseases. *Morb Mortal Wkly Rep* 1998;47:70–9
3. McGregor JA, Larsson PG. King Holmes, bacterial vaginosis and women and babies. *Int J Gynecol Obstet* 1999;67(Suppl 1):S9–S11
4. Paavonen J, Mangioni C, Martin MA, Wajszczuk CP. Vaginal clindamycin and oral metronidazole for bacterial vaginosis. *Obstet Gynecol* 2000;96: 256–60
5. Hillier SL, Holmes KK. Bacterial vaginosis. In Holmes KK, Sparling PF, Mardh P-A, *et al*, eds. *Sexually Transmitted Diseases,* 3rd edn. New York: McGraw Hill, 1999:563–86
6. Agnew KJ, Hillier SL. The effect of treatment regimens vaginitis and cervicitis on vaginal colonization by lactobacilli. *Sex Transm Dis* 1995;22:269–73
7. Hillier SL, Lipinski C, Briselden AM, Eschenbach DA. Efficacy of intravaginal 0.75% metronidazole gel for the treatment of bacterial vaginosis. *Obstet Gynecol* 1993;81:963–7
8. National Committee for Clinical Laboratory Standards. *Performance Standards for Antimicrobial Susceptibility Testing; Ninth Informational Supplement.* NCCLS document M100-S9, vol 19 no 1. Wayne, PA: National Committee for Clinical Laboratory Standards, 1999
9. Sobel JD, Schmitt C, Meriwether C. Long-term follow-up of patients with bacterial vaginosis treated with oral metronidazole and topical clindamycin. *J Infect Dis* 1993;167:783–4
10. Boris J, Pahlson C, Larsson PG. Six years observation after successful treatment of bacterial vaginosis. *Infect Dis Obstet Gynecol* 1997;5:297–302
11. Ralph ED, Clarke DA. Inactivation of metronidazole by anaerobic and aerobic bacteria. *Antimicrob Agents Chemother* 1978;14:377–83
12. Bayer AS, Chow AW, Conception N, Guze LB. Susceptibility of 40 lactobacilli to six antimicrobial agents with broad gram-positive anaerobic spectra. *Antimicrob Agents Chemother* 1978;14:720–2

13. Cunningham FE, Kraus DM, Brubaker L, Fischer J. Pharmacokinetics of intravaginal metronidazole gel. *J Clin Pharmacol* 1994;34:1060–5
14. Davis B, Glover DD, Larsen B. Analysis of metronidazole penetration into vaginal fluid by reversed-phase high-performance liquid chromatography. *Am J Obstet Gynecol* 1984;149:802–3

RECEIVED 10/27/00; ACCEPTED 11/15/00

15. Livengood CH, McGregor JA, Soper DE, *et al.* Bacterial vaginosis: efficacy and safety of intravaginal metronidazole treatment. *Am J Obstet Gynecol* 1994;170:759–64
16. Livengood CH, Soper DE, Sheehan KL, *et al.* Comparison of once-daily and twice-daily dosing of 0.75% metronidazole gel in the treatment of bacterial vaginosis. *Sex Transm Dis* 1999; 26:137–42
